# An Immunoinformatic-Based In Silico Identification on the Creation of a Multiepitope-Based Vaccination Against the Nipah Virus

**DOI:** 10.1155/2024/4066641

**Published:** 2024-06-26

**Authors:** Beant Kaur, Arun Karnwal, Anu Bansal, Tabarak Malik

**Affiliations:** ^1^ School of Bioengineering and Biosciences Lovely Professional University, Phagwara, Punjab 144411, India; ^2^ Department of Biomedical Sciences Institute of Health Jimma University, Jimma, Ethiopia

**Keywords:** B cell epitope, epidemiology, epitope-based vaccine, Nipah virus, T cell epitope

## Abstract

The zoonotic viruses pose significant threats to public health. Nipah virus (NiV) is an emerging virus transmitted from bats to humans. The NiV causes severe encephalitis and acute respiratory distress syndrome, leading to high mortality rates, with fatality rates ranging from 40% to 75%. The first emergence of the disease was found in Malaysia in 1998–1999 and later in Bangladesh, Cambodia, Timor-Leste, Indonesia, Singapore, Papua New Guinea, Vietnam, Thailand, India, and other South and Southeast Asian nations. Currently, no specific vaccines or antiviral drugs are available. The potential advantages of epitope-based vaccines include their ability to elicit specific immune responses while minimizing potential side effects. The epitopes have been identified from the conserved region of viral proteins obtained from the UniProt database. The selection of conserved epitopes involves analyzing the genetic sequences of various viral strains. The present study identified two B cell epitopes, seven cytotoxic T lymphocyte (CTL) epitopes, and seven helper T lymphocyte (HTL) epitope interactions from the NiV proteomic inventory. The antigenic and physiological properties of retrieved protein were analyzed using online servers ToxinPred, VaxiJen v2.0, and AllerTOP. The final vaccine candidate has a total combined coverage range of 80.53%. The tertiary structure of the constructed vaccine was optimized, and its stability was confirmed with the help of molecular simulation. Molecular docking was performed to check the binding affinity and binding energy of the constructed vaccine with TLR-3 and TLR-5. Codon optimization was performed in the constructed vaccine within the *Escherichia coli* K12 strain, to eliminate the danger of codon bias. However, these findings must require further validation to assess their effectiveness and safety. The development of vaccines and therapeutic approaches for virus infection is an ongoing area of research, and it may take time before effective interventions are available for clinical use.

## 1. Introduction

With different factors, including a rapidly growing human population, causing an increase in animal–human contacts, shifting environmental conditions, and inadequate sanitation and control measures, India is considered one of the world's top epicenters for diseases, including zoonotic infections. Of all zoonotic infections, 70% are caused by wildlife and bats. Numerous zoonotic virus diseases, including Marburg, SARS, and Ebola, have been associated with bats [1]. Hendra virus (HeV) is another class belonging to the same genus. Nipah contagion (nee-pa) is a recently discovered virulent that causes brain damage and death of human beings. It belongs to genus *Henipavirus*, family Paramyxoviridae, and subfamily Paramyxovirinae. It is classified under order Mononegavirales [2, 3]. The Nipah virus (NiV) and HeV are the prototype species of the Paramyxoviridae family, and both viruses closely display genome organization and protein composition. The genus *Henipaviru*s included nine *Henipavirus*, the four viral isolates of Cedar virus, Gamak virus, Daeryong virus, and Langya virus and five Ghana bat virus, Mòjiāng virus, Melian virus, Denwin virus, and Angavokely virus additional species known only from nucleic acid sequence information. The HeV and NiV are known to be associated with severe and often fatal henipaviral disease, whereas the Langya virus was associated with nonfatal febrile illness in humans [4]. The neurotropic Nipah *Henipavirus* is a pleomorphic zoonotic virus. The genetic makeup of the NiV consists of a nonsegmented, single strand of negative-sense RNA enveloped in lipid bilayer genetic material. The NiV genetic material is longer than its other family members. The genome of the NiV consists of 18,252 nucleotides [5–8]. Fruit bats are the natural hosts of the contagion, especially those in the Pteropodidae family. The first NiV isolates on Tioman Island, Malaysia, were procured from colonies of *Pteropus hypomenalus* [9]. The virus origin is based on the place name, Sungai Nipah, in the state of Perak, on Malaysia's Peninsula. In 1997, the outbreaks of encephalitic illness among pig farm workers in the Kinta District, Perak, Malaysia, were documented. The same was reported in September 1998 again in Malaysia. The same epidemic was recorded in Bangladesh in 2001, 2003, and 2004, while in India, it was observed in 2001, 2007, 2018, and 2021 [5].

A domain or zone encompassing countries of South Asia and Southeast Asian nations; countries in Asia, such as Timor-Leste, India, and Malaysia; and countries in Oceania was identified as the source of the infection [10]. Since its discovery, in Asian countries such as India and Bangladesh, the fatal outcomes range from 70% to 100% and have been verified intervening in December and March [6]. While the NiV is primarily transmitted from bats to humans, it can also spread through contaminated food or person-to-person contact. The transmission path differs between nations; for instance, in Malaysia and Singapore, the transmission of the virus is via human and animal (pig) contact. However, in India and Bangladesh, the transmission route is from infected to healthy persons and from contaminated fruit (date palm fluid) to humans [11]. The first outbreak in India was documented in Siliguri in 2001. Furthermore, a substitute outbreak was found in Nadia [12]. In Kerala State's Kozhikode District, a 28-year-old man contracted a virus in May of 2018. As documented with a 91% fatality rate out of 23 cases, only 2 patients survived [13]. The World Health Organization (WHO) resource reports that the second infection was reported in Kochi in 2019. WHO plays a vital role in monitoring and responding to NiV outbreaks and providing necessary support and resources to affected regions. The NiV infection was found in northern Himachal Pradesh [14]. In 2023, a total of 11 cases were found and the death of 8 cases was reported in Bangladesh [15].

The Institute of Epidemiology and Disease Control, Dhaka, documented that five death cases were reported in Bangladesh in 2023. Recently, the death of a 35-year-old lady was reported due to a NiV infection in the district of Rajshahi, Bangladesh. Bangladesh is a mecca of the NiV [16]. Antibodies reaching the antigen were found in *Pteropus giganteus*. The NiV was classified into two genotypic types, NiV-M and NiV-B, based on their isolation regions. Isolates from Bangladesh and India are linked to genotype B, while isolates from Malaysia and Cambodia are linked to genotype M [1]. NiV-M and NiV-B were detected in *Pteropus giganteus* and *Pteropus hypomelanus*, *Pteropus lylei*, and *Pteropus vampyrus*, respectively [17–19].

The NiV poses a significant threat to the human population due to a lack of effective therapies. The vaccine development with living pathogens poses practical biohazard challenges and management risks of handling live pathogens. The best approach is to utilize fragments of the pathogen's protein to develop a vaccine candidate to trigger a protective immune response. Currently, one vaccine is licensed for HeV in horses, monoclonal antibodies established by Australia [20].

With the epitope-based vaccine design approach used to shatter the challenge of NiV infection, the most crucial factor needed for a vaccine could be providing immunity against the NiV infection [21]. The epitope-based design method is more meticulous and efficient, minimizes time consumption, and is nonhazardous compared to the expedient vaccine design method obtainable in trade [22]. The final vaccine construction was performed by the selected seven proteins of the NiV. Immunogenic responses, three-dimensional (3D) protein modeling to better understand the designed vaccine candidate, experimental as well as computational validation, and the binding strength were determined by molecular docking. In humans, the major histocompatibility complex (MHC) alleles are commonly known as human leukocyte antigens (HLAs) with polymorphic genes [23]. Various studies have revealed that the MHC-I and MHC-II epitopes passed the selection and testing potential criteria by being antigenic (capable of eliciting immune response), nonallergenic, nontoxic, and distinct from other human proteins. The forecast epitope analysis was also performed to ensure that the constructed vaccine would be effective for a diverse population.

## 2. Methodology

### 2.1. Protein Sequence Retrieval and Analysis of the NiV Proteins

The UniProt server was used for the selection of the NiV protein. The capacity to interconnect and store the expanding universe of proteins is vital for contemporary research in life science. The ability to connect and preserve the vast universe of proteins is crucial for modern life science research, as it provides a comprehensive database of protein information. The UniProt is a great resource for biological researchers. For biologists and scientists working in functional proteomics and genomics research, the UniProt is a primary resource for protein sequences and functional interpretation [24]. The capacity of virulent proteins to infect hosts and cause disease makes them essential. The antigenic property of selected proteins was predicted using the VaxiJen v2.0 server. The parameter for the prediction set as the threshold value is 0.4 for the virus, and the antigenic score value must be greater than 0.5. Furthermore, the AllerTOP service is a valuable tool for assessing the allergenicity of proteins. AllerTOP server employs advanced algorithms to analyze protein sequences and identify potential allergens, providing crucial information about the efficacy and safety of vaccine candidates. AllerTOP v2.0 checks the allergenicity of proteins with accuracy levels at 88.7% [25–27].

### 2.2. Epitope Prediction

#### 2.2.1. Linear B Lymphocyte (LBL) Epitope Prediction

The IEBD B cell epitope prediction system was used for predicting selected proteins for B cell peptides. B cell surface receptor molecules produce antigen-related immunoglobulins. BepiPred2.0 is a machine-learning technique to detect linear B cell epitopes (LBL) with 75% accuracy [28, 29]. The other parameters for the expected B cell epitopes were determined using the AllerTOP v2.0, ToxinPred, and VaxiJen v2.0 servers [25, 30].

#### 2.2.2. Cytotoxic T Lymphocyte (CTL) Epitope Prediction

Rational vaccine design has advanced significantly with the discovery of CTL epitopes. Notably, it offers a more efficient and cost-effective alternative to traditional wet laboratory tests for predicting epitopes [31]. This was achieved by utilizing the Immune Epitope Database (IEDB)'s major histocompatibility binding tool for predicting CTL epitopes. The percentile rank which indicates the strength of affinity was set at two. The epitope selection was based on highly antigenic, nontoxic, and nonallergic, for vaccine development [32]. The online servers ToxinPred, VaxiJen v2.0, and AllerTOP v.2.0 were used to evaluate the selected epitopes' toxicity, antigenicity, and allergenicity, respectively, with an accuracy of 88.9% approximately [30].

#### 2.2.3. Helper T Lymphocyte (HTL) Epitope Prediction

HTL cells are essential for adaptive immunity because they promote B cell generation of antibodies, macrophages' absorption of infectious agents, and CTL cell destruction of target parasitized cells. Incorporating HTL cell epitopes in vaccine development is imperative to elicit an immune response. The immune cell is activated by cytokine cells. These cells include interleukin-4 (IL-4) and interleukin-10 (IL-10), as well as interferon-gamma (IFN-gamma) [33, 34]. For this reason, identifying HTL epitope cells required to promote the production of cytokines is an important step in the production of vaccines. Furthermore, the IFN-epitope server can be used with motif scan and SVM techniques [32, 35]. The IL4Pred website is used for selected peptides with the set of threshold value 2.0 which is important for subunit vaccines. The inducing qualities can also be predicted using the IL-10Pred server. The threshold value is set at 0.3 [36, 37].

### 2.3. NiV Multiepitope Vaccine (MEV) Construct

The NiV vaccine was constructed by combining the CTL, HTL, and LBL epitopes with linkers like EAAAK, AAY, GPGPG, and KK, respectively. An adjuvant is required for the construction of the best vaccine candidate. Therefore, the manufactured vaccine should be more immunogenic and enhance the immune response. The constructed vaccine used 50s ribosomal RNA L7/L12 as an adjuvant, and the fusion protein His (HHHHHH) was added at the end of the constructed vaccine [38, 39].

### 2.4. Structural Evaluation of the Developed Vaccine

Initially, comparisons were conducted using the BLASTp tool between the developed vaccine and the human proteome to assess their similarities [40]. The ProtParam web server was used to predict the physiochemical properties of the constructed vaccine [41, 42]. Furthermore, the IEDB immunogenicity tool and VaxiJen were utilized to determine the immunogenicity and antigenicity of the vaccine construct. To ascertain whether the vaccine design was allergenic, the AllerTOP server was also used [43, 44].

### 2.5. Secondary Structure Prediction of NiV Constructed Vaccine

The developed vaccination epitope's secondary structure was predicted by the PSIPRED and SOPMA tool. The PSIPRED tool, an online method for predicting the input structure, illustrated the result in graphical form, enabling easier visualization of the distribution of secondary structure in proteins. The SOPMA is an upgraded version of SOPM. The SOPMA provided the result in a score form including the variables utilized for prediction, such as window size and number of states. The first step of structure prediction is visualization proceeding with analyzing the score curve for all the projected states [45, 46]. The detailed prediction output from PSIPRED and SOPMA included alpha helix, beta-strand, coil/turn, and other structural details with 69.5% accuracy of the SOPMA result. The SoluProt tool was used in the final step to check the solubility of the constructed vaccine, which has an accuracy of 74% in a 10-fold cross-validation. [27, 47–49].

### 2.6. 3D Structure Prediction, Refinement, and Validation of NiV Vaccine

The NiV 3D protein model prediction was performed by the online software I-TASSER (Iterative Threading ASSEmbly Refinement). The SCRATCH 3Dpro tool is used for the 3D modeling of the NiV vaccine. I-TASEER server predicted the fast and accurate protein structure. The user community has offered feedback and ideas for enhancements, despite the server being extensively used in many biological and medical studies [50, 51]. After predicting the vaccine model, the Galaxy Refine web server was used for refining the constructed vaccine. The Galaxy Refine web server was used to reconfigure the side chain, structural disassembly, and overall structural inspection via molecular dynamics [52]. The Ramachandran plot has long been a primary tool for assessing protein structure validity. Since its inception, its intricate design has undergone extensive analysis and refinement, particularly with the advent of high and ultra-high-resolution experimental models of protein 3D structures. The validation software has had to be updated to account for these subtle distinctions, rendering a single Ramachandran plot insufficient for accurately delineating “favorable” and “disallowed” regions for all amino acid types [53, 54]. The Ramachandran plot was constructed using the PROCHECK server. ProSA's predicted the *Z*-score that tests the protein resemblance with native and natural structures. ERRAT is employed to check the anticipated tertiary structure of proteins [55].

### 2.7. NiV Vaccine's B Cell Epitope Prediction

MEV was predicted using the IEDB v2.22 ElliPro tool and ABCpred web server. Epitope identification is essential for vaccine development and immunotherapies, especially during a quick pandemic response [56, 57]. The computational tools help with epitope prediction, including epitope3D, a cutting-edge, scalable machine-learning technique that can precisely identify conformational epitopes. The ABCpred server with a threshold value 0.5 was used to construct the vaccine [58].

### 2.8. Disulfide Bond Prediction of MEV Protein

The disulfide bonds are covalently formed when sulfur atoms form cysteine pairs in protein structures. It helps in protein structure stability. The Protein Data Bank's high-resolution structures were used to train the neural network. The outcomes demonstrate that 99% of natural disulfide bonds can be recognized using the method suggested by Gao et al., in their studies [59]. The stability of the model vaccination is enhanced by employing a rational method for forming disulfide bonds. Proteins' geometric conformation is strengthened by disulfide bonds, which also significantly increase stability. Using the online tool DbD2 (http://cptweb.cpt.wayne.edu/DbD2/), these linkages were established. The server determined two residues having the potential to bind with each other by disulfide bond when any single amino acid changes to cysteine. Reactant pairs possessed proper geometry and disulfide bond–forming ability. If a protein contains four or more disulfide bonds, it concludes that the vaccination is stable.

### 2.9. Molecular Docking of Proteins

The efficient immunological response induced by the vaccination upon engaging the host immune system was analyzed using the PatchDock, HawkDock, and ClusPro 2.0 servers to study protein-protein docking of MEVs. Molecular docking is a fundamental step in generating the possible structure interaction and predicting binding affinity between the target vaccine candidate and the human Toll-like receptor and MHC molecules. The crystal structure of Toll-like receptors, namely, TLR-5 and TLR-3, and MHC were downloaded from the RCSB protein data bank. The PDB IDs for the TLR-5, TLR-3, and MHC-II HLA-DRB3∗01 : 01 are 8JZU, 6SWS, and 3C5J used in the ClusPro 2.0 server for docking along with NiV MEV as ligand and receptor inputs. The ClusPro 2.0 server results in the energy expressions with some correlation functions. The ClusPro 2.0 server results in the 10 best docked complex models. The cluster size must be large with low-energy docked structures [60]. Docking analysis checks the attachment ability of the constructed vaccine with Toll-like receptors and MHC molecules [61, 62].

### 2.10. Analysis of Global Population Coverage

The population coverage of projected restriction-based peptides was estimated by using the IEBD tool [63]. The MHC molecules are known as HLA molecules. The MHC-I and MHC-II classes are used for the analysis of population coverage worldwide aiming toward designing vaccines. The HLA class I is primarily associated with CTLs. The three different types of calculation were done for population coverage, namely, (1) class I separate, (2) class II separate, and (3) class I and class II combined. The histogram is generated for the percentage distribution of alleles. A cumulative coverage distribution plot is also generated to determine the minimum number of epitope/HLA combinations recognized by 90% of the population (PC90) [63]. The global distribution of HLA alleles must be considered when developing a multiepitope vaccination, as different ethnic groups exhibit varying frequencies of distinct HLA alleles and their expression. The population coverage analysis determined the suitability of selected vaccine candidates with sizeable populations [64–66].

## 3. Results

### 3.1. NiV Protein Retrieval and Analysis

All these factors have contributed to the strain selection for the interesting study. UniProtKB is a freely accessible database that contains a large information on proteins derived from the research literature. In this study, a total of eight proteins, namely, glycoprotein, fusion glycoprotein, phosphoprotein, matrix, nucleoprotein, nonstructural protein V, and protein W and C, were retrieved. Sequences of these proteins were saved in the FASTA format for the subsequent sequential investigation. Further, all the chosen proteins' antigenicity and allergic potential were determined. VaxiJen v2.0 web server was used to determine the antigenicity. The threshold value for the virus is 0.40. VaxiJen is the first server for alignment-independent prediction of protective antigens. It was developed to allow antigen classification solely based on the physicochemical properties of proteins without recourse to sequence alignment. VaxiJen v2.0 predicts the antigenic peptides with 85%–90% accuracy results. In the VaxiJen v2.0 web server, the target organism selected was the virus, and the protein sequences in plain format were used as input. ProtParam computes various physicochemical properties that can be deduced from a protein sequence. No additional information is required about the protein under consideration. The protein is an antigen; then, the protein can induce immunity in our body. The theoretical protrusion index (PI) plays an important role in the selection of protein for the further process of vaccine constructions. The GRAVY (grand average of hydropathy) score plays an important role in the epitope selection for vaccine production. Its score defines the solubility of water effect. The negative value protein is nonpolar (hydrophobic), and the positive value is polar (hydrophilic). The prediction of various values was conducted using online software. The high aliphatic index value determines the thermostability of the protein. If the instability index (II) value is more than 40, then the protein will be unstable and less than 40 means that the protein is stable where II denotes the estimated protein stability experimentally. The obtained result shown in Tables 1 and 2 found that the seven protein genes can be considered for vaccine construction. The protein C is unstable and nonantigenic and cannot produce disease.

### 3.2. Epitope Prediction

#### 3.2.1. LBL Epitope Prediction

The seven NiV proteins selected were subjected to a BepiPred Linear Epitope Prediction 2.0 using the IEBD server (Table 3). The objective was to determine capable B cell epitopes for a vaccine. The B cell's surface and immunogenicity were tested and bound to the B cell using the IEDB's antigenicity approach. The choice of epitope is essential for effective vaccine design. An epitope that is 550 amino acids long is ideal for use in vaccine production. The selected B cell epitope is then subjected to tests for toxicity (ToxinPred), allergenicity (AllerTOP v2.0), and antigenicity (VaxiJen v2.0). The ungapped epitopes, minimum identity, and peptide length proteins used to identify B cell epitopes can be modified easily by the user for further predictions and constructions of vaccine candidates [67]. The 10 B cell epitopes were found suitable after passing the selection criteria out of 34 B cell epitopes (lengths ranging from 8 to 50). The protein V epitope has both allergic and antigenic characteristics; hence, these proteins are excluded. The protein V epitope has both allergic and antigenic characteristics; hence, these proteins are excluded.

#### 3.2.2. Epitope CTL Prediction

The IEDB T cell epitope prediction used T cell epitope prediction. A T cell receptor recognized peptide-based antigen-derived molecule found on the cell surface of APCS by the MHC molecules. Two hundred peptides were obtained using the NetMHCPan EL 4.1, of which it was determined that they were MHC-specific ligands. The conserved sequences of the MHC-I class were chosen and identified (Table 4). The epitopes specifically tested opposite to MHC-I supertype alleles, including the HLA-A∗01:01, HLA-A∗02:01, HLA-A∗03:01, HLA-A∗24:02∗, HLA-A∗30:01, HLA-A∗68:01, and HLA-B∗07:02. Only 10–20 epitopes were selected from each protein for the prediction. The selected proteins were considered for the selection criteria by the high value of antigenicity, nonallergenicity, and nontoxicity during the epitope filtering procedure. Therefore, the entire epitope was not used for the vaccine construction; epitopes with a high antigenic value were selected for the vaccine construction. An epitope with a high antigenic score induces the immune response.

#### 3.2.3. HTL Epitope Prediction

The IEBD tool is used for the prediction of MHC-II epitopes. The epitopes exhibited a high affinity to MHC class II alleles along with the epitopes and their corresponding bindings to MHC class II alleles.  Table 5 shows the homology of human epitopes discovered using the NCBI BLASTp web server. UniProt/Swiss-Prot (Swiss-Prot) is the database that is utilized for forecasting, and *Homo sapiens* (taxid 9606) is the chosen organism. The *E* value indicates how many predicted hits of equivalent quality (scoring) would be discovered by chance. Because the target epitope asks for it, particularly homology to humans, the *E* (expect) value is less than 0.05. Because we will choose a nonhomologous epitope for vaccine development, the analysis of antigenicity, IL4 and IL10 induction potential, toxicity, and allergenicity served as the main foundation of selected epitopes. The MHC-II epitopes with the best profiles were chosen for further examination based on the selection criteria. Only 70 peptides passed the selection criteria, out of 2193 epitopes selected with the selected reference alleles.

#### 3.2.4. Cytokines Producing Capability

The cytokine capacity prediction is just for MHC-II. Only the helper T cell can produce cytokines during the immune response; this step is especially important for vaccine design. An online prediction server called IFN epitope attempts to identify the peptides. The selected peptide is responsible for CD4+ T cells to release IFN-gamma (a significant impact on the antiviral response). The IFN epitope was identified using in silico platforms such as IF4Pred and IF10Pred. Only 22 peptides pass the selection criteria out of 2193 epitopes.

### 3.3. Developing a Vaccine Construct

**Figure 1:**
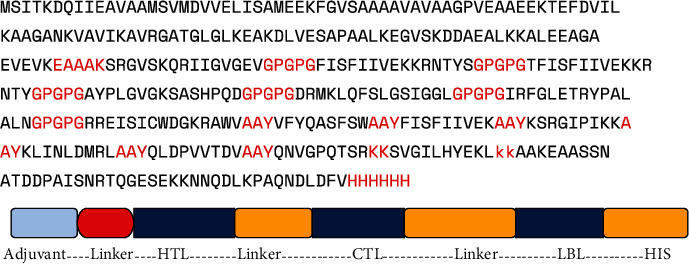
Vaccine construct.

To develop a MEV, the selected epitopes are connected by linkers such as the (B cell) KK linker, (HTL) AAY linker, and (CTL) GPGPG linker. Using the EAAAK (alpha helicase) linker, one adjuvant's 50s ribosomal RNA L7/L12 joined to create the MEV candidate [38]. A fusion protein (HHHHHH) His-tag was also introduced to the NiV construct to increase stability. The adjuvant is recognized to play important roles in strengthening immunogenic reactions like the natural immunologic response and increasing the immunogenicity of vaccines [39]. The vaccination consists of 395 amino acids, and the subunit vaccine candidate epitope with antigenic score 1 is used to construct the vaccine. The antigenic score of HTL is considered above 0.5. The EAAAK linker maintained the distance to improve the protein stability (Figure 1).

### 3.4. Identify the Physiochemical Properties of the Developed Vaccine

The ProtParam server predicted the physiochemical characteristics of the created NiV vaccine. These metrics provide vital information about the stability, solubility, and other characteristics of the vaccine design. Additionally, the antigenicity and allergic potential predated servers are used to predict these parameters (Table 6). To use protein-sol software to determine the solubility of a manufactured vaccine candidate, the expected solubility is represented by the scaled solubility value (QuerySol). The value of the solubility must be greater than 0.45. This is due to the PopAvrSol, checking for the probability of a solution using the SoluProt v2.0 server [48, 68].

### 3.5. Establishing the Secondary Structure of a NiV Vaccine

**Figure 2:**
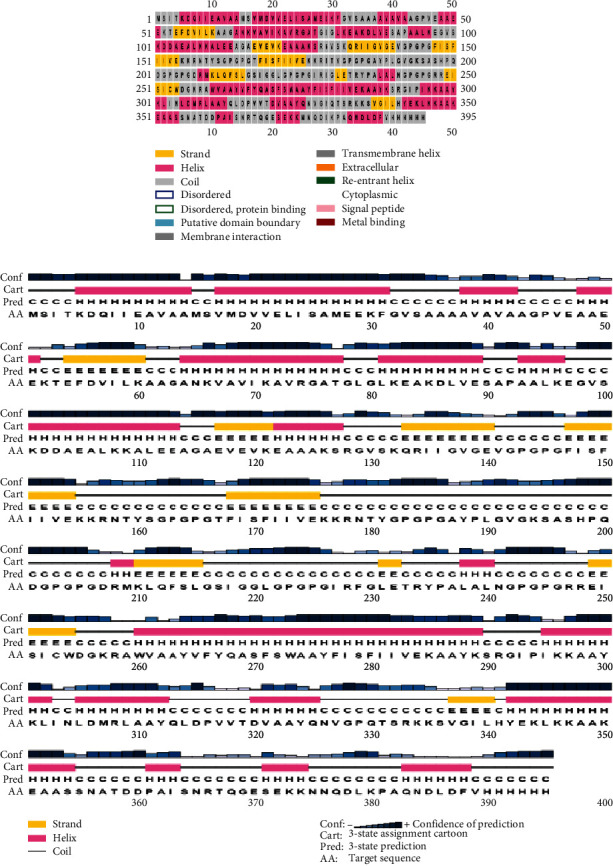
Anticipated Nipah virus secondary conformation: (a) visual representation of PSIPRED's result page; (b) graphical representation of expected secondary structure features.

The SOPMA predicts three distinct properties of a NiV secondary structure: the creation of a coil/turn structure in a built vaccine epitope, an alpha helix, and a beta strand (Figure 2). The outcomes of predicting an epitope's secondary structure using PSIPRED are represented graphically. The 395 amino acid residue sequences were uploaded on the SOPMA server to the built-in vaccination. The length of the coils is 35.70%, beta sheets are 6.58%, alpha helix is 34.48%, and extended stars are 19.24%, respectively.

**Figure 3:**
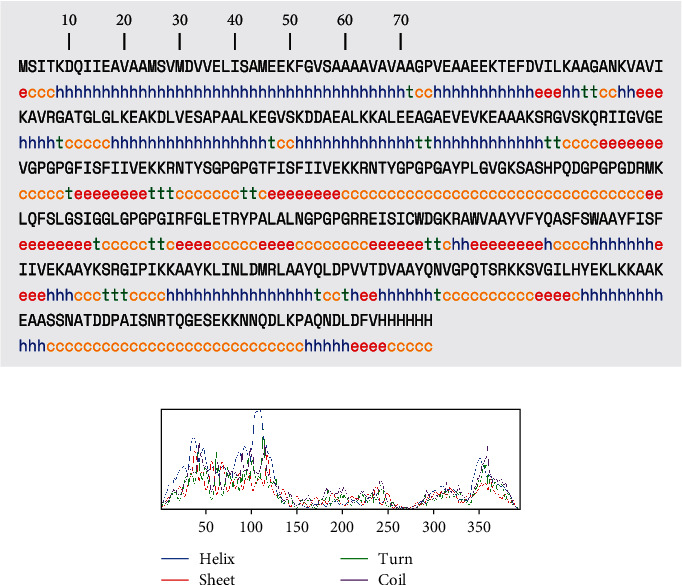
The SOPMA result page showing two graphs: (a) the first illustrates the prediction step; (b) the second displays the predicted score curves.

The SOPMA result page (Figure 3) has two graphs displayed. The first step is depicted in the prediction. In the second step, the score curves are predicted. Additionally, it displays the parameters used in the prediction, such as the number of states and window width. An attachment containing the text version of the prediction result file is provided. Links are provided to access the intermediate result files as well.

### 3.6. Tertiary Structure Prediction, Refinement, and Validation of Constructed NiV

**Figure 4:**
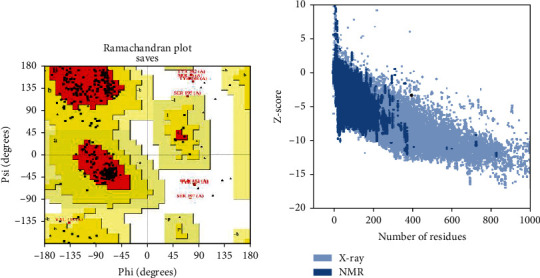
(a) The Ramachandran plot of Nipah virus multiepitope vaccine. (b) *z*-score value plot of Nipah virus multiepitope vaccine.

The 3D structure of constructed NiV MEV candidate predicted by I-TASSER server. The server is used for protein modeling/molecular modeling. The predicted 3D model has a *C*-score of −1.01 and an RMSD value of 9.1 ± 4.6 Å. The Galaxy Refine server was used for the refinement of predicted 3D model for Rama favored by side chain repacking of the candidate. The Ramachandra plot is predicated on the PROCHECK server. The PROCHECK server checks the stereochemical quality of a protein structure by analyzing residue-by-residue geometry and other structure geometry. About 86.4% of residues have been in the most favored regions, 11.2% residues have been found in the additional allowed regions, 0.6% residues in generously allowed regions, and 1.8% residues in disallowed regions. Based on the analysis of 118 structures of resolution of at least 2.0 Å and *R*-factor is less than 20%, the model would be expected to be as good as 90% in the most favored region. The NiV MEV candidate tertiary model has 40 glycine residues (triangles in Figure 4) and 22 proline residues. The *z*-score value of the constructed NiV vaccine candidate was −3.36.

### 3.7. NiV Vaccine's B Cell Epitope Prediction

**Figure 5:**
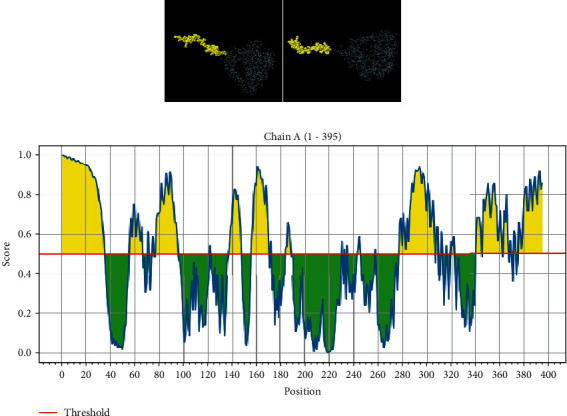
(a) 3D mapping structure of continuous and discontinuous epitopes. (b) 2D score chart of the constructed multiepitope vaccine candidate of Nipah virus continuous and discourteous epitopes.

The IEDB v2.22 ElliPro tool is used to predict linear and discontinuous antibodies based on the geometrical properties of protein structure (Figure 5(a)) [69]. It forms the structures of antibody-protein complexes. The NiV MEV has 11 linear epitopes with the highest PI value of 0.899 scores of 89.9% including 36 residues and a discontinuous linear epitope score of 0.943, which means 94.3% including 31 residues (Figure 5(b)).

### 3.8. MEV Protein Disulfide Bond Prediction

**Figure 6:**
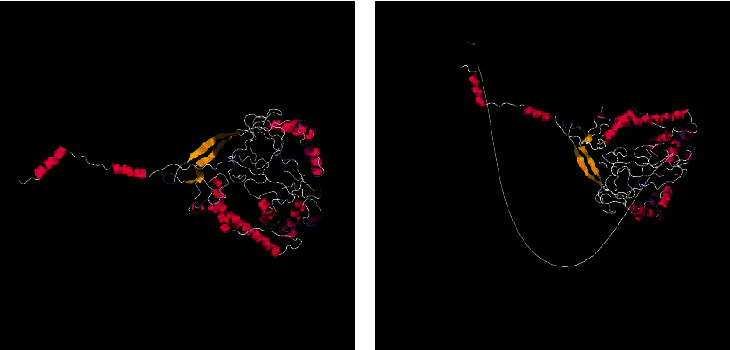
(a) The 3D structure of the constructed vaccine candidate. (b) The 3D structure of disulfide bond formation after mutation in seven residues of chain A and chain B.

The DbD2 server was used to predict the disulfide bond of two residues of proteins of constructed vaccine candidates. The PDB model is used for the prediction of disulfide bonds. The disulfide bond enhances the protein stability, protein function, and dynamics. The constructed vaccine candidate can be used if the mutation occurs between the two residues (Figure 6). The analysis runs based on residue pair information including energy, angle, *B*-factor, torsion angle, a bond value between the two-residue chain of the vaccine candidate, and beta-factor value. The torsion angle ranges from +125.95 to −65.97, and the bond is 1.38–10.25 kcal/mol. The change in seven residues showed the disulfide bond formation.

### 3.9. Molecular Docking of Proteins

**Figure 7:**
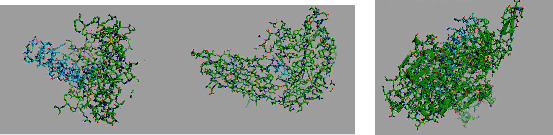
Molecular docking of Nipah virus multiepitope vaccine candidate with (a) TLR3 and TLR5 and (b) MHC-II.

The molecular docking of the Nipah vaccine multiepitope candidate analysis with the ClusPro 2.0 web tool is online. The vaccine candidate acts as the receptor molecule and is docked with the TLR3 (PDB:7C76), TLR5 (PDB:3J0A), and the MHC class-II allele HLA-DRB3∗01:01 (PDB:3C5J) as shown in Figure 7. The 30 models were generated for each docking. Following the molecular docking, 30 models were generated for each of the docking candidates. The NiV MEV binds with TLR3 (PDB:7C76) with a binding energy of −674.4 kcal/mol; with TLR5 (PDB:3J0A), the binding affinity was −553.0 kcal/mol; and with MHC class-II allele HLA-DRB3∗01:01 (PDB:3C5J), the interaction was −824.2 kcal/mol.

### 3.10. Population Coverage of NiV

**Figure 8:**
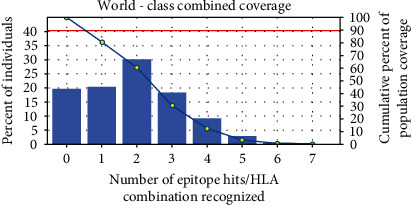
The combined world population coverage of constructed vaccine.

The epitopes of MHC-I and MHC-II have strong binders used to predict the global population (Figure 8). These were found to be (Table 4) epitopes that were effective MHC-I binders. Similar to this, there were (Table 5) epitopes identified as powerful MHC-II binders. Finally, the epitopes with the higher antigenic score value of 1.0 were considered for the prospective NiV design along with other filtering parameters like interferon-potential, antigenicity, nonallergenicity, and nontoxicity. The final NiV was constructed from 16 epitopes in total. The NiV shared a 100% sequence similarity with all 16 epitopes. The population coverage analysis program analyzed the epitope population coverage study; KPKLISYTL, MPKSRGIPI, KLINLDMRL, TPMPKSRGI, KSRGIPIKK, QLDPVVTDV, and QNVGPQTSR are MHC-I (CD8 T cell) epitopes. The world population coverage for the seven MHC-II (CD4+T cell) epitopes is as follows: SRGVSKQRIIGVGEV, FISFIIVEKKRNTYS, TFISFIIVEKK RNTY, AYPLGVGKSASHPQD, DRMKLQFSLGSIGGLG, IRFGLETRYPALALN, and RREISICWDGKRAWV, respectively. The coverage range is 61.81% and 17.84%, and the combined coverage range is 80.53% and 62.68%, respectively, within the world.

## 4. Discussion

Although there are licensed vaccines and medications to treat NiV infections, the struggle is frequently one-sided and these diseases have always been devastating. A subunit vaccine candidate against the NiV infections has been sought for using immunoinformatic analysis. To build a possible MEV candidate for the Nipah vaccination, we used a reliable immunoinformatic technique together with in vivo experiments in this work. The initial step of the study was to identify CD4+ epitope peptides that would bind to MHC-II. Furthermore, conserved epitopes were refined and found that all predicted epitopes are expected to be peptide-inducing interferons. Moreover, a constructed vaccine passed all selection criteria required to develop a suitable vaccine. The final constructed vaccine has 395 amino acid residues. The NiV and TLR-3 and MHC-II receptors formed a stable complex. The results of the molecular docking showed that the developed vaccine binds effectively with MHC-II molecule with a binding affinity of −990.6 kcal/mol. The resulting multiepitope NiV vaccine is highly immunogenic, safe, and nontoxic, based on our computational research results. These outcomes unequivocally demonstrate that the immunogen used in this work was capable of promoting antibody synthesis in vivo.

## 5. Conclusion

NiV polyprotein has been targeted using computational tools to identify T cell and B cell epitopes which have been applied to develop a NiV MEV in this study, and the designed NiV MEV construct is expected to have antigenicity and nonallergic potential. A highly selective structure of protein was created, showing markable population coverage. The T lymphocytes (CTLs), the T lymphocytes (HTLs), and the B lymphocytes (LBLs) were tested and selected for the design of vaccines. The constructed vaccines showed a high antigenicity and nonallergenicity. The final vaccines are composed of B and T cell epitopes with the help of linkers and adjuvants. Analysis of the conservation and population coverage of promiscuous epitopes revealed a robust immune response to the NiV—molecular docking with human TLR-3 and TLR-5 results in a cellular response confirmed by molecular dynamic simulations. Our computational analysis suggests that the vaccine manufactured is highly immunogenic, safe, nontoxic, and stable. The candidate NiV- MEV designed in this study could be further developed via various in vitro and in vivo studies to determine its preliminary safety and efficacy.

## Figures and Tables

**Table 1:** The antigenic potential of Nipah virus protein.

**Protein name**	**Length (amino acid)**	**Entry name in UniProt**	**Accession ID**	**Gene**	**VaxiJen score**
Glycoprotein G	602	GLYCP_NIPAV	Q91H62	G	0.511 (antigenic)
Fusion glycoprotein F0	546	FUS_NIPAV	Q91H63	F0	0.5012 (antigenic)
Matrix protein	352	MATRX_NIPAV	Q91K90	M	0.4033 (antigenic)
Phosphoprotein	709	PHOSP_NIPAV	Q91K91	P	0.5866 (antigenic)
Nucleoprotein N	532	NCAP_NIPAV	Q91K92	N	0.5713 (antigenic)
Protein W	450	W_NIPAV	P0C1C7	W	0.6279 (antigenic)
Protein C	166	C_NIPAV	Q997F1	C	0.33 (nonantigenic)
Nonstructural protein V	456	V_NIPAV	Q997F2	V	0.6252 (antigenic)

**Table 2:** Identification allergenic characteristics, antigenicity, GRAVY scores, and PI scores of Nipah virus proteins.

**Protein name**	**Accession ID**	**Gene**	**VaxiJen score**	**GRAVY score**	**Aliphatic index**	**Instability index**	**Theoretical PI**
Glycoprotein G	Q91H62	G	0.511 (antigenic)	−0.178	90.95	34.56 (stable)	8.58
Fusion glycoprotein Fo	Q91H63	F0	0.5012 (antigenic)	0.195	112.44	38 (stable)	5.85
Matrix protein	Q91K90	M	0.4033 (antigenic)	−0.211	90.26	29.53 (stable)	9.31
Phosphoprotein	Q91K91	P	0.5866 (antigenic)	−0.73	76.29	48.52 (unstable)	4.61
Nucleoprotein N	Q91K92	N	0.5713 (antigenic)	−0.236	86.28	52.33 (unstable)	6.06
Protein W	P0C1C7	W	0.6279 (antigenic)	−0.826	67.78	57.07 (unstable)	4.84
Protein C	Q997F1	C	0.33 (nonantigenic)	−0.345	86.87	43.67 (unstable)	9.44
Nonstructural protein V	Q997F2	V	0.6252 (antigenic)	−0.816	65.61	60.47 (unstable)	4.66

**Table 3:** Predicted epitopes obtained from BepiPred Linear Epitope Prediction 2.0 server along with VaxiJen analysis showing the epitopes' antigenicity, allergenicity, and toxicity.

**Protein name (no. of amino acids)**	**Accession ID**	**Initial**	**Stop**	**Peptide**	**Length**	**Antigenicity**	**Value**	**Allergenicity**	**Toxicity**
Glycoprotein G (602)	Q91H62	8	43	VRFENTTSDKGKIPSKVIKSYYGTMDIKKINEGLLD	36	A	0.5116	NA	NT
Fusion glycoprotein F0 (546)	Q91H63	24	33	SVGILHYEKL	10	A	1.3889	NA	NT
		523	543	NTYSRLEDRRVRPTSSGDLYY	21	A	0.7837	NA	NT
Matrix protein (352)	Q91K90	5	41	IKSISSESMEGVSDFSPSSWEHGGYLDKVEPEIDENG	37	A	0.5324	NA	NT
		245	254	RAGKYYSVDY	10	A	0.5617	NA	NT
Phosphoprotein (709)	Q91K91	238	258	YTSDDEEADQLEFEDEFAGSS	21	A	0.6049	NA	NT
Nucleoprotein (532)	Q91K92	109	130	NGIPVMERRGDKAQEEMEGLMR	22	A	0.5319	NA	NT
		133	158	KTARDSSKGKTPFVDSRAYGLRITDM	26	A	0.8649	NA	NT
		487	528	AAKEAASSNATDDPAISNRTQGESEKKNNQDLKPAQNDLDFV	42	A	1.0342	NA	NT
Protein W (450)	P0C1C7	237	258	LYTSDDEEADQLEFEDEFAGSS	22	A	0.5206	NA	NT

**Table 4:** Result showing an antigenic score, allergenicity, toxicity, and SVM score of MHC-I epitope.

**Protein**	**Allele**	**Start**	**End**	**Peptide**	**Antigenicity**	**Antigenic score**	**Allergenicity**	**Toxicity**	**SVM score**
Glycoprotein G (602)	HLA-B∗07:02	19	27	KPKLISYTL	A	1.0819	NA	NT	−1.28
	HLA-A∗01:01	43	51	AMDEGYFAY	A	0.7221	NA	NT	−0.83
	HLA-A∗03:01	15	23	RLSIGSPSK	A	0.7713	NA	NT	−1.04
	HLA-A∗24:02	32	40	VFYQASFSW	A	1.0092	NA	NT	−1.33
	HLA-B∗07:02	49	57	RPKLFAVKI	A	0.5410	NA	NT	−0.82

Fusion glycoprotein F0 (546)	HLA-A∗03:01	47	55	KIKSNPLTK	A	0.725	NA	NT	−1.35
	HLA-A∗03:01	32	40	KLSKIGLVK	A	0.5490	NA	NT	−0.47
	HLA-A∗01:01	18	26	SLDLALSKY	A	0.8317	NA	NT	−0.94
	HLA-A∗68:01	14	22	SVMENYKTR	A	0.4955	NA	NT	−0.25
	HLA-A∗68:01	32	40	FISFIIVEK	A	1.1861	NA	NT	−0.79

Phosphoprotein (709)	HLA-A∗30:01	38	46	KSRGIPIKK	A	1.397	NA	NT	−1.04
	HLA-B∗07:02	36	44	MPKSRGIPI	A	1.4672	NA	NT	−1.21
	HLA-A∗02:01	45	53	KLINLDMRL	A	1.9291	NA	NT	−1.29
	HLA-B∗07:02	34	42	TPMPKSRGI	A	1.3217	NA	NT	−1.08
	HLA-A∗03:01	38	46	KSRGIPIKK	A	1.397	NA	NT	−1.04
	HLA-A∗02:01	46	54	QLDPVVTDV	A	1.2435	NA	NT	−1.24
	HLA-A∗30:01	51	59	SIRDNLQAK	A	1.0609	NA	NT	−0.45

Nucleoprotein (532)	HLA-B∗07:02	5	13	GPRAPYMVL	A	0.4773	NA	NT	−1.04
	HLA-B∗07:02	23	31	RPGALIRSL	A	0.6313	NA	NT	−0.39

Protein W (450)	HLA-A∗30:01	38	46	KSRGIPIKK	A	1.397	NA	NT	−1.04
	HLA-B∗07:02	36	44	MPKSRGIPI	A	1.4672	NA	NT	−1.21
	HLA-B∗07:02	34	42	TPMPKSRGI	A	1.3217	NA	NT	−1.08
	HLA-A∗03:01	38	46	KSRGIPIKK	A	1.397	NA	NT	−1.04
	HLA-A∗02:01	46	54	QLDPVVTDV	A	1.2435	NA	NT	−1.24
	HLA-A∗30:01	51	59	SIRDNLQAK	A	1.0609	NA	NT	−0.45

Protein C (166)	HLA-A∗02:01	1	9	MMASILLTL	A	0.5423	NA	NT	−1.6
	HLA-A∗68:01	16	24	QTMGMYVLY	A	0.5194	NA	NT	−0.55
	HLA-A∗01:01	16	24	QTMGMYVLY	A	0.5194	NA	NT	−0.55

Nonstructural protein V (456)	HLA-A∗30:01	38	46	KSRGIPIKK	A	1.397	NA	NT	−1.04
	HLA-B∗07:02	34	42	TPMPKSRGI	A	1.3217	NA	NT	−1.08
	HLA-A∗03:01	38	46	KSRGIPIKK	A	1.397	NA	NT	−1.04
	HLA-A∗02:01	46	54	QLDPVVTDV	A	1.2435	NA	NT	−1.24
	HLA-A∗30:01	51	59	SIRDNLQAK	A	1.0609	NA	NT	−0.45
	HLA-A∗03:01	51	59	SIRDNLQAK	A	1.0609	NA	NT	−0.45
	HLA-A∗68:01	42	50	EIAVSKEDR	A	0.9943	NA	NT	−1.25
	HLA-A∗68:01	40	48	QNVGPQTSR	A	1.1044	NA	NT	−0.92

**Table 5:** List of MHC-II epitopes predicted showing highlighted selected epitopes with high affinity towards MHC-II.

	**Allele**	**Peptide**	**Antigenicity score**	**Antigenicity**	**Allergenicity**	**Toxicity**	**Homology**
Glycoprotein G (602)	HLA-DRB3∗01:01	ASFSWDTMIKFGDVL	0.9791	A	NA	NT	NH
	**HLA-DRB3**∗**01:01**	**QASFSWDTMIKFGDV**	**1.1926**	**A**	**NA**	**NT**	**NH**
	HLA-DRB3∗01:01	YQASFSWDTMIKFGD	0.9929	A	NA	NT	NH
	HLA-DRB4∗01:01	VIIVMNIMIIQNYTR	0.4392	A	NA	NT	NH
	HLA-DRB4∗01:01	IVMNIMIIQNYTRST	0.4581	A	NA	NT	NH
	**HLA-DRB4**∗**01:01**	**SRGVSKQRIIGVGEV**	**1.0135**	**A**	**NA**	**NT**	**NH**
	HLA-DRB3∗01:01	VYNDAFLIDRINWIS	0.5724	A	NA	NT	NH
	HLA-DRB3∗01:01	VFYQASFSWDTMIKF	0.4522	A	NA	NT	NH
	HLA-DRB3∗01:01	FYQASFSWDTMIKFG	0.8327	A	NA	NT	Nh
	HLA-DRB1∗15:01	IVMNIMIIQNYTRST	0.4527	A	NA	NT	NH
	HLA-DRB3∗01:01	SFSWDTMIKFGDVLT	0.8178	A	NA	NT	NH
	HLA-DRB3∗01:01	FSWDTMIKFGDVLTV	0.8494	A	NA	NT	NH
	HLA-DRB1∗15:01	VMNIMIIQNYTRSTD	0.5664	A	NA	NT	NH
	HLA-DRB1∗15:01	MNIMIIQNYTRSTDN	0.5675	A	NA	NT	NH

Fusion Glycoprotein F0(546)	HLA-DRB3∗01:01	DLALSKYLSDLLFVF	0.4441	A	NA	NT	NH
	HLA-DRB1∗03:01	HDLVGDVRLAGVIMA	0.4319	A	NA	NT	NH
	**HLA-DRB1**∗**07:01**	**AVVKLQETAEKTVYV**	**0.5491**	**A**	**NA**	**NT**	**NH**
	**HLA-DRB5**∗**01:01**	**RNTYSRLEDRRVRPT**	**0.5681**	**A**	**NA**	**NT**	**NH**
	**HLA-DRB1**∗**15:01**	**SEWISIVPNFILVRN**	**0.5788**	**A**	**NA**	**NT**	**NH**
	**HLA-DRB5**∗**01:01**	**NTYSRLEDRRVRPTS**	**0.8149**	**A**	**NA**	**NT**	**NH**
	**HLA-DRB1**∗**03:01**	**ISFIIVEKKRNTYSR**	**1.229**	**A**	**NA**	**NT**	**NH**
	**HLA-DRB5**∗**01:01**	**GLITFISFIIVEKKR**	**1.4212**	**A**	**NA**	**NT**	**NH**
	**HLA-DRB1**∗**03:01**	**FISFIIVEKKRNTYS**	**1.4463**	**A**	**NA**	**NT**	**NH**
	**HLA-DRB5**∗**01:01**	**TFISFIIVEKKRNTY**	**1.4822**	**A**	**NA**	**NT**	**NH**
	**HLA-DRB1**∗**07:01**	**NDNSEWISIVPNFIL**	**0.5496**	**A**	**NA**	**NT**	**NH**
	HLA-DRB1∗15:01	IGLITFISFIIVEKK	1.4477	A	NA	NT	NH
	HLA-DRB1∗15:01	LCIGLITFISFIIVE	1.2143	A	NA	NT	NH

Matrix Protein (352)	HLA-DRB3∗01:01	MLEFRRNNAIAFNLL	1.1537	A	NA	NT	NH
	HLA-DRB3∗01:01	TMLEFRRNNAIAFNL	1.2843	A	NA	NT	NH
	**HLA-DRB1**∗**07:01**	**AYPLGVGKSASHPQD**	**1.0442**	**A**	**NA**	**NT**	**NH**
	**HLA-DRB4**∗**01:01**	**DRMKLQFSLGSIGGL**	**1.0588**	**A**	**NA**	**NT**	**NH**
	HLA-DRB1∗15:01	KRKKIRTIAAYPLGV	0.4099	A	NA	NT	NH

Phosphoprotein (709)	HLA-DRB3∗01:01	QSLFSFDNVKNFRDG	0.5702	A	NA	NT	NH
	HLA-DRB3∗01:01	SLFSFDNVKNFRDGS	0.6857	A	NA	NT	NH
	HLA-DRB1∗03:01	**GNVCLVSDAKMLSYA**	**0.7717**	A	NA	NT	NH
	HLA-DRB1∗03:01	NVCLVSDAKMLSYAP	0.8356	A	NA	NT	NH
	HLA-DRB1∗03:01	NNGNVCLVSDAKMLS	0.6279	A	NA	NT	NH
	HLA-DRB1∗03:01	VCLVSDAKMLSYAPE	0.8236	A	NA	NT	NH
	HLA-DRB1∗03:01	NSIKLINLDMRLNHI	1.5884	A	NA	NT	NH
	HLA-DRB3∗01:01	LEQQSLFSFDNVKNF	0.5997	A	NA	NT	NH
	HLA-DRB3∗02:02	AENVQLNASTAVKET	0.6499	A	NA	NT	NH
	HLA-DRB3∗01:01	LFSFDNVKNFRDGSL	0.8488	A	NA	NT	NH
	HLA-DRB1∗03:01	CLVSDAKMLSYAPEI	0.7505	A	NA	NT	NH

Nucleoprotein (532)	HLA-DRB3∗01:01	DIEAVIIDVGSMVNG	0.6401	A	NA	NT	NH
	**HLA-DRB1**∗**15:01**	**IRFGLETRYPALALN**	**1.4919**	**A**	**NA**	**NT**	**NH**
	HLA-DRB1∗07:01	YPLLWSFAMGVATTI	0.7452	A	NA	NT	NH

Protein W (450)	HLA-DRB1∗03:01	GNVCLVSDAKMLSYA	0.7717	A	NA	NT	NH
	HLA-DRB1∗03:01	NVCLVSDAKMLSYAP	0.8356	A	NA	NT	NH
	HLA-DRB1∗03:01	NGNVCLVSDAKMLSY	0.5498	A	NA	NT	NH
	**HLA-DRB1**∗**15:01**	**PSSVGGKPNESIGRT**	**0.6845**	**A**	**NA**	**NT**	**NH**
	HLA-DRB1∗03:01	NNGNVCLVSDAKMLS	0.6279	A	NA	NT	NH
	HLA-DRB1∗03:01	VCLVSDAKMLSYAPE	0.8236	A	NA	NT	NH
	HLA-DRB1∗03:01	CLVSDAKMLSYAPEI	0.7505	A	NA	NT	NH
	HLA-DRB3∗01:01	DKLELVNDGLNIIDF	0.8133	A	NA	NT	NH

Non- structural Protein V (456)	HLA-DRB1∗03:01	GNVCLVSDAKMLSYA	0.7717	A	NA	NT	NH
	HLA-DRB1∗03:01	NVCLVSDAKMLSYAP	0.8356	A	NA	NT	NH
	**HLA-DRB1**∗**03:01**	**RREISICWDGKRAWV**	**1.1002**	**A**	**NA**	**NT**	**NH**
	**HLA-DRB1**∗**15:01**	**PSSVGGKPNESIGRT**	**0.6845**	**A**	**NA**	**NT**	**NH**
	**HLA-DRB1**∗**07:01**	**GKRVSNTRDWAEGSD**	**0.4377**	**A**	**NA**	**NT**	**NH**
	**HLA-DRB1**∗**07:01**	**IGKRVSNTRDWAEGS**	**0.5191**	**A**	**NA**	**NT**	**NH**
	**HLA-DRB1**∗**07:01**	**TIGKRVSNTRDWAEG**	**0.465**	**A**	**NA**	**NT**	**NH**
	**HLA-DRB5**∗**01:01**	**ADRQRPGTPMPKSRG**	**0.6577**	**A**	**NA**	**NT**	**NH**
	**HLA-DRB5**∗**01:01**	**TADRQRPGTPMPKSR**	**0.5585**	**A**	**NA**	**NT**	**NH**
	HLA-DRB1∗03:01	NGNVCLVSDAKMLSY	0.5498	A	NA	NT	NH
	HLA-DRB1∗03:01	NNGNVCLVSDAKMLS	0.6279	A	NA	NT	NH
	HLA-DRB1∗03:01	VCLVSDAKMLSYAPE	0.8236	A	NA	NT	NH
	HLA-DRB1∗03:01	CLVSDAKMLSYAPEI	0.7505	A	NA	NT	NH
	HLA-DRB3∗01:01	DKLELVNDGLNIIDF	0.8133	A	NA	NT	NH
	HLA-DRB1∗03:01	LVSDAKMLSYAPEIA	0.698	A	NA	NT	NH

**Table 6:** Physiochemical properties of the developed vaccine.

**Vaccine construction characteristics**	
Vaccine length	395
Molecular weight	41892.94
Antigenicity	0.7560
Allergic potential	Nonallergenic
Toxicity	Nontoxic
Theoretical PI	9.11
Instability index	25.73
Total atom number	5946
AI	85.32
Extinction coefficient	34380
GRAVY score	−0.194
Solubility	0.865

## Data Availability

Data will be available at particular and acceptable request.
